# Impact of three different plate colours on short-term satiety and energy intake: a randomized controlled trial

**DOI:** 10.1186/s12937-018-0350-1

**Published:** 2018-04-21

**Authors:** Asli Akyol, Aylin Ayaz, Elif Inan-Eroglu, Cansu Cetin, Gulhan Samur

**Affiliations:** Department of Nutrition and Dietetics, Hacettepe University, Sihhiye, 06100 Ankara, Turkey

**Keywords:** Colour, Plate, Food, Energy intake, Satiety response

## Abstract

**Background:**

Plate colour was previously shown to alter the amount of food consumption due to its environmental effect on food intake. However, different studies have indicated that the effect of plate colour cannot be generalized. In light of this finding, the main objective of this study was to determine whether food consumption during an open buffet meal was different when using same-sized white, red or black plates.

**Methods:**

This study was a crossover study conducted with 54 female participants aged 18–30 years with normal BMIs. On experimental days, participants ate a standard breakfast and were then randomly assigned to eat ad libitum lunch (pasta with tomato sauce and soft drinks) using white, red or black plates. Visual analogue scale (VAS) scores on satiety outcomes were measured for all meals. Energy and macronutrient intake during lunch was recorded.

**Results:**

The results showed that plate colour exerted a significant effect on food intake during the test days (*p* = 0.021). The average total energy intake with red (1102.16 ± 47.12 kcal, *p* = 0.05) and black plates (1113.19 ± 47.12 kcal, *p* = 0.034) was significantly increased when compared to that with white plates (945.72 ± 47.12 kcal). There were no differences between red and black plates (*p* = 0.985). Overall, mean VAS scores did not indicate a significant difference between the groups.

**Conclusions:**

Plate colour may be a crucial determinant of energy intake.

## Background

There is strong interest in identifying environmental factors linked with overeating because improvement of those factors might prevent or reverse obesity [1]. Over the last 30 years, eating patterns have changed, and the number of meals eaten outside the home, commercial portion sizes, and dishware sizes have increased both at home and outside the home [2, 3]. These alarming trends in the food environment may have contributed to difficulty in restraining food intake and thus increased prevalence of overweight and obesity as a result of increased energy consumption. The need for efficient strategies in restraining food intake is evident as self-regulation of appetite and appetitive behaviour, which depend on several factors, becomes difficult in modern societies [4].

Several studies reported that modifications in food environments may alter eating patterns [5, 6]. For instance, environmental factors, such as the presence of other people [7], location [8], portion size [9] and time of consumption [10], appear to affect food intake and food choice. More specifically, the colour of the plate ware, of the packaging, and of the surrounding environment has been shown to influence food consumption [11]. In light of these outcomes, assessing the environmental and situational colour-related cues that induce inhibitory reactions towards food intake can be beneficial to improve and regulate healthy eating habits.

Recent studies showed that red can be a cognitive and behavioural stimulus in humans due to its high visibility and its perception as a signal of danger [12, 13]. The observations originating from these studies introduced the hypothesis that using red plates may elicit similar avoidance behaviour towards food intake. Genschow et al. [14] reported that individuals drank and ate less from red labelled cups and red plates than from blue labelled cups and blue and white plates. Similarly, another study demonstrated reduced consumption of popcorn and chocolate chips when the foods were served on red plates [15]. However, other studies reported that red plate was not always a valid predictor of food intake [16]. Instead, the reaction towards the intake of food from different coloured plates developed through several adaptations. One of these adaptations depended on the perceived healthiness of the food, as Reutner et al., [17] indicated that the impact of red colour decreased when food options became healthier. Second, the contrast between the plate colour and the food colour appeared to be an important factor [16]. van Ittersum and Wansink [16] reported that contrast, instead of the specific colour of the plate, was the main factor in restricting food intake. Since the effect of plate colour on food intake remains unresolved in these studies, there is a need for further studies with different types of foods and contrasts [14, 15].

In the present study, the main aim was to examine energy intake in the context of three different plate colours (white, red and black) during an open buffet meal. The open buffet meal consisted of pasta and non-carbonated soft drinks, which were consumed during a regular meal time since most of the studies conducted to assess the effect of plate colour on food intake were conducted with snack foods [14, 15, 18]. In addition, the current study included three different plate colours with measured colorimetric coordinates. White plates were used as the control group, and black plates were used to test the contrast difference with respect to red plates. As subjective satiety outcomes are influenced by external factors and are related to subsequent food intake, the second aim of the study was to measure participant’s satiety outcomes using the visual analogue scale (VAS) [19].

## Methods

### Subjects

Fifty-four female subjects were recruited from Hacettepe University and the surrounding community through poster advertisements and announcements. A questionnaire examining general health and nutritional habits was used for screening all volunteers. Inclusion criteria included women in good health between the ages of 18–30 years who were non-smokers, not dieting and not diagnosed with any metabolic disease. They could not be colour blind, be professional athletes, possess food allergies or extreme dislikes for specific foods or be pregnant or lactating. None of the participants were taking medications known to affect appetite or weight regulation. Regular breakfast, snack, and lunch consumption was an additional inclusion criterion for participants. Each subject signed an informed consent document before the study. Subjects having extreme dislikes for specific foods were also excluded. Subjects were excluded if they scored > 9 on a Beck depression scale due to predisposition to depression and had a measured body mass index (BMI) < 18 or > 25 kg/m^2^. Participants’ body weight, height, and body compositions in terms of lean body mass (kg) and fat percentage (%) were measured with a Jawon XScan Plus (Jawon Medical, Gyeongsang, Korea). Since the menstrual cycle may affect appetite ratings, experiment days were arranged one week before menstruation for all participants. The Non-Interventional Clinical Studies Ethics Board of Hacettepe University (GO16/751–16) provided ethical approval on the 6th of December 2016.

### Study design and experimental protocol

This study was a crossover study conducted at the Nutrition Laboratory in the Department of Nutrition and Dietetics, Hacettepe University, Ankara, Turkey. The experimental protocol of this study used the European consensus on postprandial studies evaluating appetite measures and eating behaviours [20]. The study was carried out on three separate days, with one to two weeks washout period between each study day. On each test day, participants were randomly assigned to eat lunch using a plastic white, red or black plate. A randomization scheme was generated using the website “randomization.com” to allocate participants to the different plate colours [21]. Each plate colour was named W, R and B in the randomization system, and then, plates were served to the corresponding subject according to the randomization letters by an investigator who was not involved in the trials. Participants were informed that the topic of the research was to examine their energy intake on different test days.

On experimental days, participants arrived at 08.00 h after fasting for 12 h and left at 14.00 h. On the previous evening, participants were guided to have dinner, namely, a bowl of tomato soup (200 mL), grilled meat (60 g), salad (200 g), white bread (50 g) (total, 602 kcal) and adequate water, and on the following morning, they were served breakfast, namely, two thin slices of white bread (50 g), a slice of cheese (30 g), and a cup of tea at 08.30 h (total, 250 kcal). Participants were asked to consume the full breakfast within 15 min. Additional food or beverage intake was not allowed until the open buffet lunch. At 12.00 h, an ad libitum buffet-style lunch was served. Lunch consisted of pasta and soft drinks. The recipe for the pasta was as follows: 500 g pasta was boiled in hot water with 50 g sunflower oil. Then, pasta was added to 300 g ready-to-eat Napolitana sauce and mixed until homogenously distributed. The nutritional value of 100 g of this recipe was 287 kcal, 44.8 g carbohydrates, 8.0 g protein and 8.2 g fat. The soft drinks were served in their original containers in order to simulate a typical situation and assess the effect of plate colour alone. The nutritional value of 100 mL of fruit juices (orange, peach and mixed) was between 63 and 90 kcal, with 12.7–20.0 g carbohydrates.

The serving table was a separate dining table at a moderately close distance where the subjects sat. On each test day, the same amounts and types of foods were served, and the buffet items were identical. The same portion sizes, serving cutlery, and serving bowls were used on the test days. The subjects were instructed to have lunch until comfortably satisfied and allowed to refill their plate whenever they wanted.

Throughout the study, participants were in the same room and were allowed to read or use laptops throughout the experiment. Physical activity and social interaction were limited. Subjects were not allowed to see how much other subjects consumed. Energy and macronutrient intakes of subjects were measured by weighing the amounts of food and drink consumed and converting these values into energy (kcal) and macronutrients based on the manufacturer’s labelling.

### VAS

The VAS was used to assess hunger, satiety, prospective food consumption, amount of food they could consume, and desire for sugary foods throughout the study period [22]. Appetite ratings were recorded on a 100 mm visual analogue scale (VAS) with words anchored at each end describing the extremes of a unipolar question (for hunger: “I am not hungry at all”/“I have never been more hungry”; for satiety: “I am not sated at all”/“I have never been more sated”; for prospective food consumption: “I cannot consume any food at all”/“I have never wanted to consume food that much”; for desire for a sugary snack: “I do not want to consume a sugary snack at all”/“I have never wanted to consume a sugary snack that much”; for amount of food: “I can only have a small amount of food”/“I can eat a large amount of food”). The baseline VAS scores were measured before breakfast at 08.00 am. Then, participants were asked to consume breakfast until 08.30. After breakfast, participants filled in VAS questionnaires every 15 min until lunch and afterwards for a total of 23 times. Before the study period, subjects were informed as to how to fill out VAS forms.

### Photometric measurements

The colorimetric coordinates of the plates used in the study are shown in Table 1. The measurements were performed with a Datacolor Mercury ™ 2000 Color Measurement System under a Xenon pulsed lamp with a spectral gap of 400–700 nm, a measuring range of 0–200% reflection and a bandwidth efficiency/resolution of 100 nm/2 nm. The colour difference equation was used according to CIE2000.

**Table 1:** Colorimetric coordinates of the white, red and black plates used in the study

	L	a	b	c	H
White	88.88	−1.37	−2.44	2.79	240.73
Red	47.76	0.28	−1.46	1.49	280.99
Black	30.50	45.94	25.83	52.7	29.35

L: The L dimension of the space is roughly proportional to log-luminance and captures perceived changes along the black–white achromatic colour continuum. a, b: The a and b dimensions define a chromatic plane with axes corresponding to the red–green and blue–yellow opponent continua. c: Chroma is the perceived strength of a surface colour and the degree of visual difference from a neutral grey of the same lightness. H: Hue is the attribute of appearance that is often colloquially called colour

### Statistical analysis

Data were analysed using the Statistical Package for the Social Sciences (SPSS) version 22 (SPSS Inc., Chicago, IL, USA). The primary outcome of this trial was the effect of different coloured plates on energy intakes during an ad libitum lunch. For the primary outcome, data were analysed using a general linear model and ANOVA. The secondary outcome variables were subjects’ VAS scores. For the secondary outcome, data were analysed using a repeated-measures ANCOVA with baseline measurement as the covariate. Subjects and test day were included in the procedure, in addition to the plate colours/time interaction. Bonferroni post hoc analysis was used for comparisons between black and red plates. Data for the area under the curve (AUC) for VAS scores were obtained using GraphPad Prism version 6 (GraphPad Software Inc., La Jolla, CA, USA). Baseline values were added as covariates in AUC data. Data are given as the mean ± standard error of mean unless otherwise stated. *P* < 0.05 was considered statistically significant. Power analysis indicated that at least 24 subjects per condition were required in order to estimate a minimum effect size of 18.35% (for energy intake differences) for comparisons between treatment arms (yielding a power of 0.80 and alpha of 0.05) [23].

## Results

All of the participants completed the study successfully. All subject data were analysed for each test meal. Participants were 22.22 ± 0.14 years of age, with an average BMI of 21.29 ± 0.12 kg/m^2^, and had a waist circumference of 70.09 ± 0.48 cm. Body composition data for the participants were 44.19 ± 0.31 kg lean body mass and 23.91 ± 0.33% fat.

**Fig. 1:**
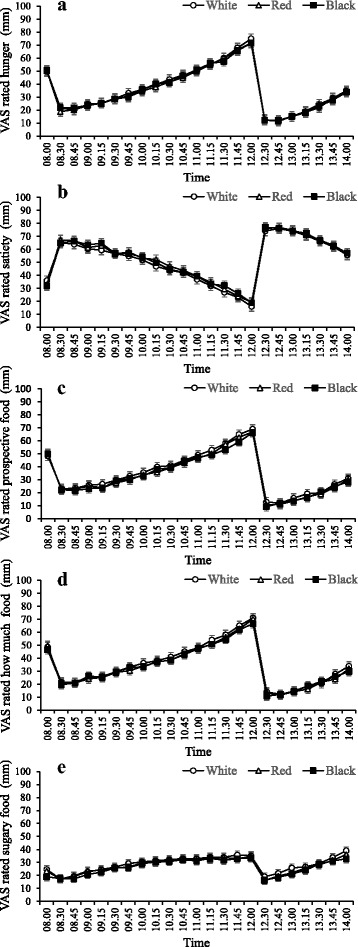
Mean VAS scores (± SEM) during the three test days, *n* = 54. (**a**) VAS-rated hunger, (**b**) VAS-rated satiety, (**c**) VAS-rated prospective food consumption, (**d**) VAS-rated amount of food that could be consumed, (**e**) VAS-rated desire for a sugary snack. A light breakfast was served at 08.00 h, immediately after recording baseline VAS scores. Lunch was served at 12.00 h. Repeated measures indicated that there were no statistically significant differences between white, red and black plates (*P* > 0.05)

Figure 1 shows the mean VAS-rated hunger, satiety, prospective food consumption, amount of food that could be consumed, and desire for sugary foods for the total study period. Baseline values did not differ between test days (*P* > 0.05). VAS scores indicated that both breakfast and lunch significantly influenced VAS scores (*P* < 0.001). However, plate colour did not affect VAS scores during the test days (P > 0.05). No interaction was detected between plate colour and time. In addition, AUC data of VAS scores did not show a significant difference between the groups (Table 2) (*P* > 0.05).

**Table 2:** Area under curve (AUC) data for VAS scores

VAS questions	Plate colours		
	White	Red	Black
Hunger	217.4 ± 25.3	212.4 ± 25.1	214.0 ± 24.6
Satiety	303.7 ± 28.7	320.80 ± 29.3	320.2 ± 29.7
Prospective consumption	215.5 ± 21.2	201.9 ± 20.6	200.0 ± 20.4
Amount of food that could be consumed	215.7 ± 24.8	203.1 ± 23.6	203.8 ± 23.7
Desire for sugary snack	174.3 ± 19.1	164.7 ± 17.4	159.2 ± 16.5

Mean VAS scores (± SEM) during the three test days (*n* = 54). There were no statistically significant differences between treatments (*P* > 0.05)

Energy intakes from pasta and drink during the open buffet lunch are shown in Table 3. Participants were allowed to refill their plates by visiting the serving table as much as they wanted in the present study. To distinguish exact calories between multiple visits to the buffet, the energy intake of each participants was separately recorded for refilled plates (2nd visit). At the first visit, participants’ energy intake from pasta was significantly increased with red and black plates when compared to that with white plates (*P* = 0.025). The energy intake from soft drinks was similar for all plate colours (*P* > 0.05). Overall, the data indicated that the total energy intake (pasta and drink) of participants at the first visit to the buffet was significantly higher with red and black plates than with white plates (*P* = 0.03) (Table 3). Thirteen participants with white plates, sixteen participants with red plates and fifteen participants with black plates visited the buffet a second time, and participants did not have additional drinks. At this stage of the study, plate colours did not exert a significant effect on energy intake (P > 0.05) (Table 3). None of the participants visited the buffet a third time. When the total energy intake was evaluated, using red and black plates resulted in higher energy intake than using white plates (*P* = 0.021) (Table 3). Energy intake from red and black plates was similar.

**Table 3:** Energy intake during the open buffet lunch meal

	Plate colours								
Energy intake (kcal)	White (Pasta energy)	White (Drink energy)	White (Total energy)	Red (Pasta energy)	Red (Drink energy)	Red (Total energy)	Black (Pasta energy)	Black (Drink energy)	Black (Total energy)
1st visit	740.67 ± 28.84	100.02 ± 15.28	850.70 ± 33.81	842.79 ± 28.84§	90.35 ± 15.32	957.89 ± 33.81*	837.29 ± 28.84§	96.33 ± 15.65	964.97 ± 33.81*
2nd visit	337.89 ± 69.26	–	337.89 ± 69.26	402.38 ± 62.43	–	402.38 ± 62.43	514.93 ± 64.48	–	514.93 ± 64.48
Total	902.65 ± 30.24	100.02 ± 15.28	947.72 ± 47.12	1084.45 ± 32.85‡	90.35 ± 15.32	1102.16 ± 47.12†	1095 ± 36.20‡	96.33 ± 15.65	1113.19 ± 47.12†

Mean energy intake scores (± SEM) during the three test days (*n* = 54). All participants visited the serving table for the 1st round with white, red or black plates (n = 54). Data on the 2nd visits to the serving table consisted of *n* = 13 participants for white plates, *n* = 16 participants for red plates, and *n* = 15 participants for black plates. § indicates the significant effect of plate colour on energy intake from pasta at the first visit to the serving Table (*P* = 0.025). * indicates the significant effect of plate colour on energy intake from pasta and drink at the first visit to the serving Table (*P* = 0.03). ‡ indicates the significant effect of plate colour on energy intake from pasta in total (*P* = 0.028). † indicates the significant effect of plate colour on energy intake from pasta and drink in total (*P* = 0.021). Post hoc tests did not show a significant difference between red and black plates (*P* > 0.05)

## Discussion

The present study examined whether or not three different colours (white, red or black) of plates would exert a significant effect on short-term satiety and food intake. Interestingly, for the first time, the results of this study demonstrated that energy intake from red and black plates was significantly increased when compared to white plates in women of a normal weight who were allowed to eat freely during lunch. With respect to VAS scores, subjective reporting of satiety-related parameters was similar between the three plate colours. Although red was considered to evoke danger- and stop-related signals towards food intake in different studies [14, 15], our results suggested the possible role of additional factors that could be related to colour cues and food consumption.

Most of the studies that evaluated the effect of plate colour on food intake used rather snacks due to their independent effect on total energy intake [15, 18]. Since the composition and quantity of the main meals constitute a crucial part of daily energy intake [24, 25], the current study was particularly interested in the effect of plate colour on regular meals that occur on a daily basis. Therefore, the open buffet meal in the present study consisted of pasta with tomato sauce as a healthy and commonly consumed food. Our results showed that red plate colour did not reduce food intake compared to white plate. In addition to this effect, the presence of healthy food resulted in the consumption of larger amounts of food without any inhibitory effect. Notably, this impact was observed during only the first visit to the serving table. The initial food consumption might have compromised the further influence of having more food during a regular meal time, or simply, this effect might have been lessened over consumption time of the meal. As discussed by Genschow et al. [14], the link between the red plate and amount of energy intake from healthy or unhealthy foods was an open question, and in line with their speculation, this link appeared to be dependent on the type of food. Hence, in light of our results, it can be suggested that using red plates to control food intake might be an ineffective strategy in individuals who are healthy and consuming healthy foods.

The consumption of larger amounts of food from red or black plates can be explained by the contrast hypothesis rather than the specific effect of the plate colour [15]. In a related study, van Ittersum and Wansink [16] reported that participants consumed significantly more pasta when the colour contrast was lower between the pasta and the plate. For instance, participants had larger amounts of pasta when offered red sauce on red plates or white sauce on white plates. This finding seems to be consistent with our data, although van Ittersum and Wansink reported the serving size instead of the actual energy intake [16]. Since the present study recorded food intake and consumed amounts at every visit to the buffet, it can be concluded that contrast could be an important factor for the determination of the effect of plate colour on energy intake. Although the colour coordinates of the pasta with tomato sauce were not measured in the current study due to the heterogeneous texture of the food, it was observed that the colour of the served pasta was closer to red when compared to white because of the colour of the sauce. However, given that the black plate was also associated with the consumption of higher amounts of pasta, the simpler explanation of the similarity between the red sauce and red plate may not be accurate, as the colour coordinates of red and black plates that were used in the present study were distinct. Were et al. [15] reported that the defined direction of the contrast did not exert a significant effect, as they observed similar influence of red plates on popcorn and chocolate chip consumption. Similarly, the second study of Genschow et al. [14] showed the reduced food intake in red plates, as in their study, participants consumed significantly fewer pretzels with blue or white plates. Therefore, further examinations including different types of foods and plate colours are needed to elucidate the contrast issue.

Taste expectations were shown to be mediated according to different colour plates [26]. Piqueras-Fiszman et al. [18] demonstrated that participants rated the intensity of the flavour more when the mousse sample was served on white plates in comparison to black plates. In this context, the specific effect of the colour black on food consumption is unknown. Data generated from these studies introduced interesting questions of whether the resulting perception of consumers according to different plate colours will influence the consumption of these foods. As black plates induced a higher energy intake in the present study, a possible explanation may be that black plates can make food more appealing than white plates. Genschow et al. [12] addressed this issue in a further analysis. Despite finding that red plates were liked more and were rated more attractive compared to white plates, food intake from red plates was still reduced. The present study did not examine the perception of flavour, quality, aesthetics of plates or liking for open buffet lunch. However, the VAS scores examined related parameters, such as the desire for sugary snacks or prospective food consumption, and all of these parameters were similar between the three plate colours. This result may suggest a possible direct effect of plate colours instead of an interaction with perceptive parameters. At present, how subjective perception, plate colour and food intake interact with each other is an open question. Future research may aim to investigate these connections on different occasions.

The current study has several limitations that should be addressed. First, this experiment was conducted in a laboratory where healthy participant’s usual eating habits might be affected due to the different environment. Regarding the effects of plate colour on perception in an ecologically natural setting, one study confirmed that using white or black plates resulted in significantly different perception of the food in a restaurant [27]. In addition, the health status of the participants in such studies can also determine the observed effects, as one study showed that a high contrast intervention (red) versus baseline (white) resulted in significantly higher food and drink intake in patients with severe Alzheimer’s disease [28]. Therefore, future studies may continue to perform similar research under natural settings and with different clinical groups. Second, the open buffet lunch consisted of pasta and soft drinks in the current study. It is well known that multi-item buffet meals can encourage overeating and increase food intake [29]. When the possible influence of food colour and interactions between other environmental colours and food colour is considered [30, 31], testing related hypotheses with a multi-item buffet meal can help us understand the current issues better. Third, eating behaviours are known to be influenced by sex differences [32]. Conducting similar experiments with male subjects can extend the current findings in future studies.

## Conclusions

In conclusion, the present study demonstrated that red and black plates did not induce avoidance behaviour in healthy women during lunch. The results of this study suggest that the influence of plate colour on food intake may depend on other factors, such as the type of the food and contrast interaction between the colours of the plate and the served food. As the need for efficient strategies in restricting food intake is crucial to combating overeating and increased energy intake, research assessing the impact of colour on eating behaviour should be continued in future studies.
